# Type H blood vessels in bone modeling and remodeling

**DOI:** 10.7150/thno.34126

**Published:** 2020-01-01

**Authors:** Yi Peng, Song Wu, Yusheng Li, Janet L. Crane

**Affiliations:** 1 Department of Orthopedic Surgery, The Third Xiangya Hospital of Central South University, Changsha, Hunan 410013, China.; 2 Department of Orthopedic Surgery, Xiangya Hospital of Central South University, Changsha, Hunan 410008, China; National Clinical Research Center for Geriatric Disorders, Xiangya Hospital, Central South University, Changsha, Hunan 41000, China.; 3 Department of Orthopedic Surgery, Johns Hopkins University School of Medicine, Baltimore, MD, USA; Department of Pediatrics, Johns Hopkins University School of Medicine, Baltimore, MD, USA.

**Keywords:** Type H vessels, H-type vessels, CD31^hi^Emcn^hi^ endothelial cells, Angiogenesis, Osteogenesis, Bone formation

## Abstract

In the mammalian skeletal system, osteogenesis and angiogenesis are intimately linked during bone growth and regeneration in bone modeling and during bone homeostasis in bone remodeling. Recent studies have expanded our knowledge about the molecular and cellular mechanisms responsible for coupling angiogenesis and bone formation. Type H vessels, termed such because of high expression of Endomucin (Emcn) and CD31, have recently been identified and have the ability to induce bone formation. Factors including platelet-derived growth factor type BB (PDGF-BB), slit guidance ligand 3 (SLIT3), hypoxia-inducible factor 1-alpha (HIF-1α), Notch, and vascular endothelial growth factor (VEGF) are involved in the coupling of angiogenesis and osteogenesis. This review summarizes the current understanding of signaling pathways that regulate type H vessels and how type H vessels modulate osteogenesis. Further studies dissecting the regulation and function of type H vessels will provide new insights into the role of bone vasculature in the metabolism of the skeleton. We also discuss considerations for therapeutic approaches targeting type H vessels to promote fracture healing, prevent pathological bone loss, osteonecrosis, osteoarthritis, and bone metastases.

## 1. Introduction

Osteogenesis is crucial to maintain the integrity and function of the mammalian skeletal system. Dysfunctions in osteogenesis result in various bone diseases. An imbalance of bone resorption with bone formation during bone remodeling results in osteoporosis. Inadequate bone formation during bone modeling can result in bone disorders such as malunion of fracture. Angiogenesis, the development of new blood vessels from pre-existing vessels, is intimately linked to osteogenesis during skeletal development and during bone modeling and remodeling. Blood vessels not only provide bone tissues with the necessary nutrients, oxygen, growth factors, and hormones, they have more recently been found to play an essential role in the regulation of bone formation 1, 2.

The process of long bone development involves bone modeling and coupled bone remodeling. In bone modeling, bone formation occurs independently of bone resorption, whereas bone resorption and formation are coupled during bone remodeling to maintain skeletal homeostasis 3-5. Despite their differences, both processes are coupled with angiogenesis 6. The intimate spatial and temporal link between osteogenesis and angiogenesis has been termed “angiogenic-osteogenic coupling” 7-11. For example, in bone modeling associated with endochondral ossification, hypertrophic chondrocytes express high levels of vascular endothelial growth factor (VEGF), which promotes the vascular invasion of cartilage, recruits chondroclasts to resorb hypertrophic cartilage and osteoblasts to build the bone matrix 12. In bone modeling in response to mechanical loading, macrophage/non-resorbing osteoclast lineage cells secrete platelet-derived growth factor type BB (PDGF-BB) to recruit both endothelial and osteoblast precursor cells to couple angiogenesis with osteogenesis 13.

**Figure 1:**
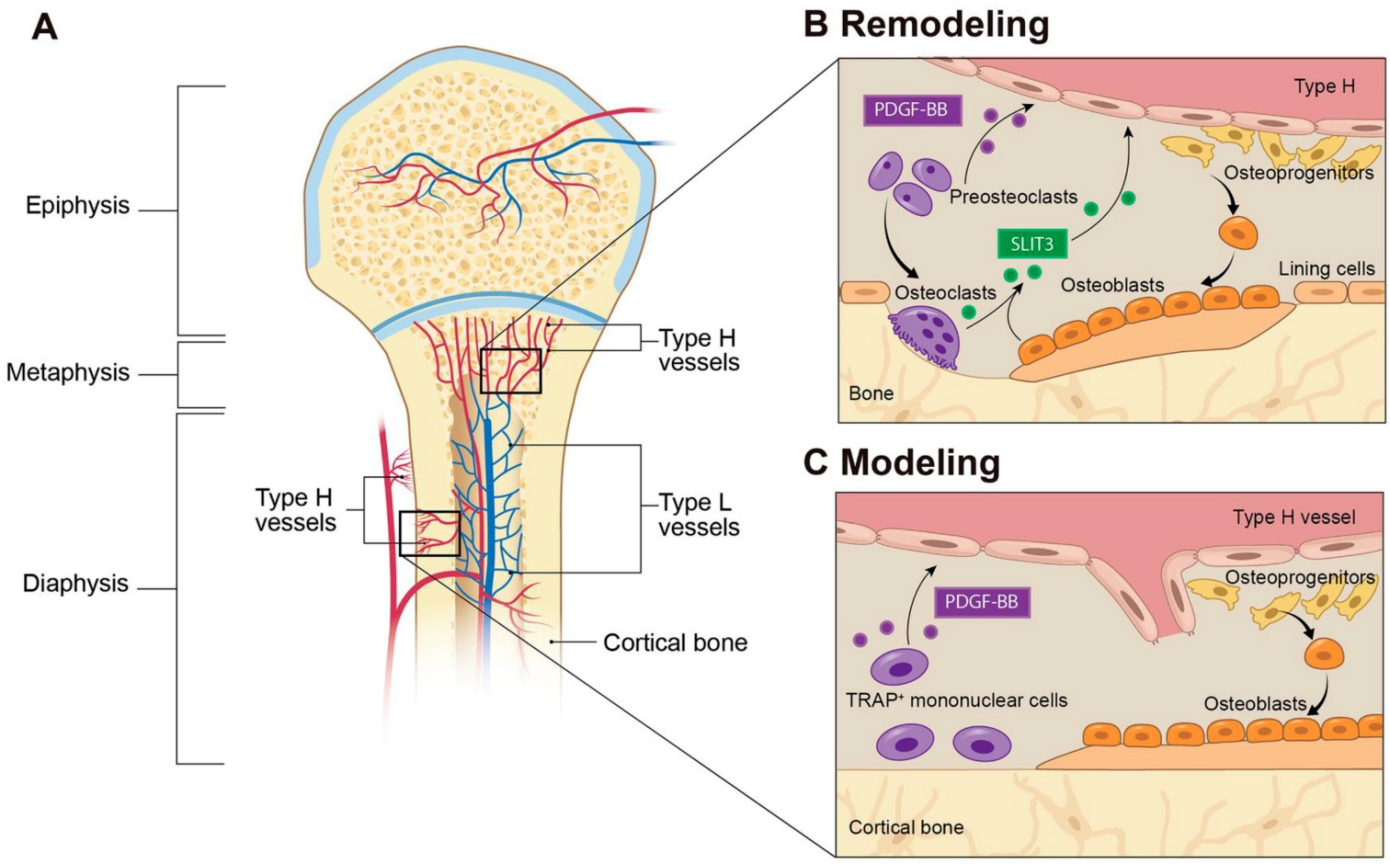
** Type H blood vessels in bone modeling and remodeling. (A)** Spatial distribution of arteries, veins, and capillaries in metaphysis and diaphysis regions of long bone. Arteries branch into smaller arterioles and connect to type H vessels which are localized near the growth plate in the metaphysis or positioned at the periosteum and endosteum proximal to compact bone. Blood then flows to Type L vessels before exiting the bone through the central vein. (**B**) In bone remodeling, preosteoclasts differentiate to bone-resorbing osteoclasts. Preosteoclasts secrete the growth factor PDGF-BB, leading to increased type H vessels formation through enhanced recruitment of ECs and assembly into vessels. PDGF-BB also recruits osteoprogenitor cells from the type H vessels to the bone remodeling site for differentiation into bone-forming osteoblasts. Mature osteoclasts and osteoblasts release SLIT3, which enhances endothelial tube formation and branching of type H vessels to couple bone resorption with bone formation. (**C**) In bone modeling, TRAP^+^ mononuclear cells secrete PDGF-BB into the periosteum, which recruits periosteal progenitor cells for both endothelial and osteoprogenitor cell differentiation to couple type H vessel formation with periosteal bone formation. Abbreviations: ECs, endothelial cells; PDGF-BB, platelet-derived growth factor type BB; SLIT3, slit homolog 3 protein; TRAP, tartrate-resistant acid phosphatase.

Knowledge about the cellular mechanisms that underlie molecular communications between endothelial cells (ECs) and osteoblast lineage cells has been rapidly expanding. A subtype of capillary that is associated with osteogenesis was recently characterized and termed type H vessels, which is defined by high expression of CD31 and Endomucin (CD31^hi^Emcn^hi^). Type H vessels are located near the growth plate in the metaphysis and both the periosteum and endosteum of the diaphysis (**Figure 1A**). Type H vessels are densely surrounded by osteoprogenitors that express the transcription factor Osterix, which is a potent promoter of bone formation. Evidence indicates the presence of molecular crosstalk from osteoblast lineage and osteoclast lineage cells to ECs to promote angiogenesis. Type H vessels can actively direct bone formation by production of factors that stimulate proliferation and differentiation of osteoprogenitors in the bone marrow 14-16.

This review discusses the regulation and role of type H vessels in osteogenesis and provides an overview of potential therapeutic approaches targeting type H vessels in various bone disorders. PDGF-BB and slit guidance ligand 3 (SLIT3) stimulate endothelial and mesenchymal cell migration to induce type H vessel and bone formation 13, 14. Hypoxia-inducible factor 1 alpha (HIF-1α), Notch, and VEGF signaling, which are well known factors in the vascular field that induce EC proliferation, migration, and vessel formation, are also expressed by osteoblasts, chondrocytes, and bone ECs to further stimulate angiogenesis. Manipulation of these factors in ECs has recently uncovered new insights into the production of osteogenic factors by ECs to directly regulate bone formation. These factors begin to explain coupling of angiogenesis and osteogenesis at the molecular level. Pharmacologically targeting type H vessels in various animal models of bone disease, such as osteoarthritis, osteoporosis, fracture healing, osteonecrosis, and bone metastases demonstrate improvement. Further clinical studies are needed to evaluate significance of type H vessels and efficacy of EC target therapies for bone disorders.

## 2. Identification of type H vessels in bone

Blood vessels are composed of multiple types of cells, among which ECs play a central regulatory role in the processes of vascularization and angiogenesis. Bone blood vessels are lined with specialized ECs that possess specific morphologic, molecular, and functional properties. Kusumbe et al. identified a special subtype of blood vessels in trabecular and cortical bone adjacent to the growth plate and along the periosteal and endosteal surface, respectively. These vessels were termed type H vessels because of high expression of CD31 and Emcn. Type H vessels are organized as straight columns interconnected at their distal end 16. In contrast, sinusoidal vessels in the diaphysis form a dense, highly branched capillary network in the bone marrow cavity of the diaphysis, which show low expression of CD31 and Emcn, termed type L vessels (**Figure 1A**). Oxygen rich blood flows from arteries and distal arterioles and connects directly to type H vessels. Blood then continues to the type L sinusoidal network at the interface between the metaphysis and diaphysis and eventually drains into the central vein.

Type H vessels have a dense arrangement of osteoprogenitors, largely expressing Osterix, which can differentiate into osteoblasts and osteocytes 17. Runx2^+^ early osteoprogenitors and collagen type 1α^+^ osteoblast cells are also densely arranged around the CD31^+^ vessels in the metaphysis and endosteum 16. In contrast, osteoprogenitors and osteoblasts are not present near the diaphyseal sinusoidal type L vessels. The close proximity of osteoprogenitors to type H vessels confers a resource of osteoblasts for bone formation, while the high oxygen content provides necessary nutrients to meet the metabolic demands of osteogenesis (**Figure 1A**).

Type H vessels are noted most abundantly in young, growing animals, adjacent to the growth plate. The elongation of long bones by the process of endochondral ossification occurs at the growth plate and involves continuous replacement of cartilage matrix by calcified bone to acquire their typical shapes through bone modeling. Cells in the metaphysis of postnatal and adolescent bone provide signals to promote type H vessels. Osteoclasts and/or chondroclasts have been thought to remove cartilage to make lacunae for blood vessel growth. Recently, Romeo et al reported that proteinases including matrix metalloproteinase-9 (MMP-9) released from type H ECs, not osteoclasts, are essential for resorbing cartilage to lead longitudinal bone growth 18. Their findings suggest that vessel-associated osteoclasts can stimulate ECs to help digest the cartilage template and thereby aids in regulating blood vessel growth during endochondral ossification.

## 3. Factors involved in coupling type H vessel formation and osteogenesis

**Figure 2:**
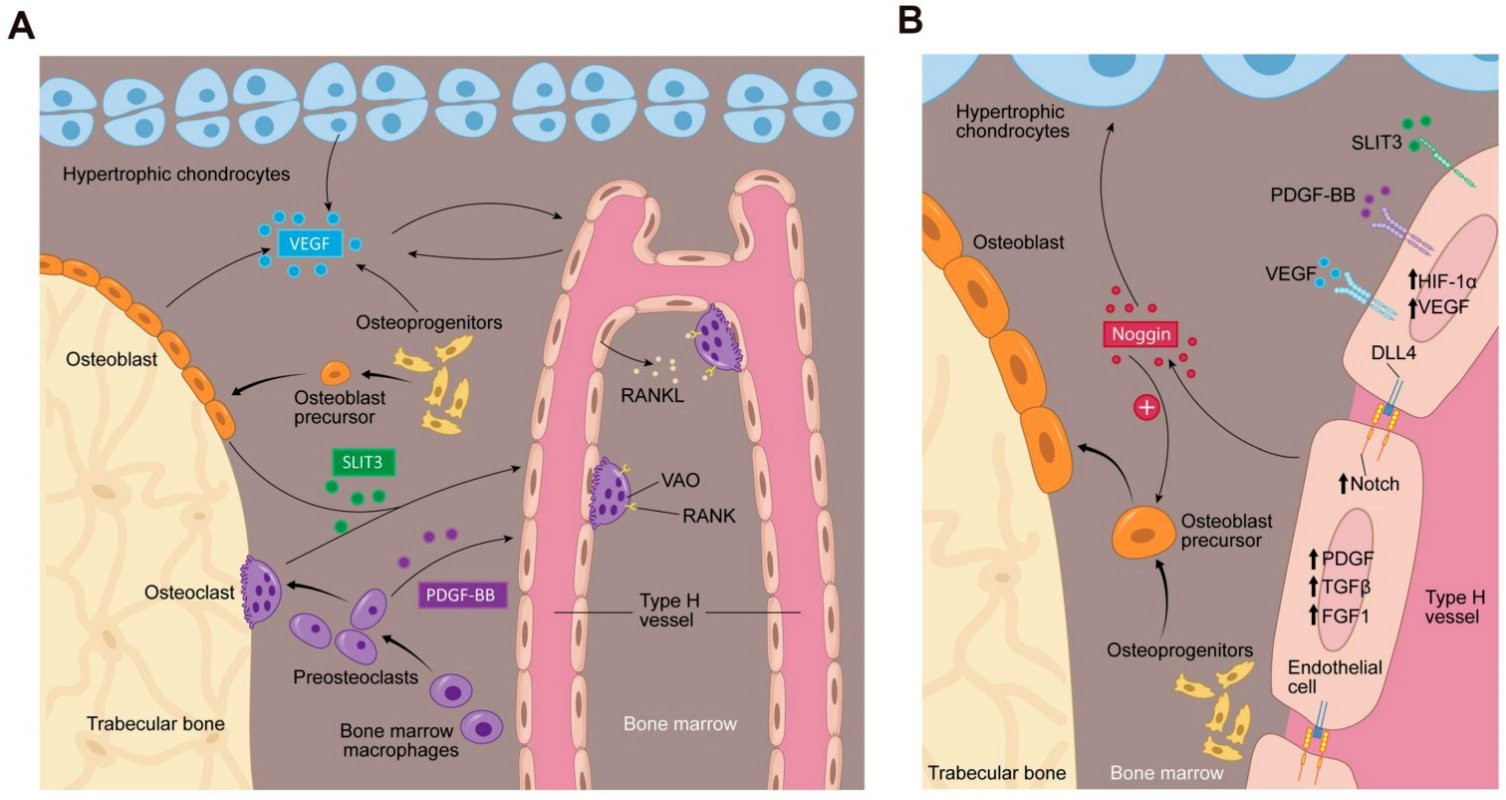
** Bone marrow microenvironment paracrine signaling coupling angiogenesis and osteogenesis. (A)** Multiple cells secrete factors into the bone marrow microenvironment to support type H vessel formation. Hypertrophic chondrocytes, osteoblast lineage cells and ECs can secrete VEGF. Mature osteoblasts and osteoclasts secrete SLIT3. Preosteoclasts secrete PDGF-BB. VEGF, SLIT3, and PDGF-BB promote type H vessels formation. Type H vessels are accompanied by osteoprogenitors and become embedded between trabecular bone in the metaphysis and extend into arches adjacent to the growth plate. Type H ECs express RANKL and support VAOs through a RANKL-RANK signaling mechanism to facilitate cartilage resorption and bone formation. VAOs also promote anastomoses of type H vessels.** (B)** In an oxygen insufficient environment, type H ECs increase HIF-1α expression, which triggers the expression of genes controlling angiogenesis, such as VEGF, similar to the mechanism of osteoblast and hypertrophic chondrocyte VEGF secretion. VEGF binds its receptor on ECs to stimulate blood vessel growth. PDGF-BB and SLIT3 also bind to their respective receptors on ECs, which can further enhance EC VEGF expression and promote EC migration, tube formation, and branching. The increase in blood flow can stimulate Notch signaling within the EC. Endothelial Notch/Dll 4 signaling stimulates Noggin production. Noggin stimulates differentiation of perivascular osteoprogenitor cells, facilitates chondrocyte hypertrophy maturation, and promotes EC proliferation. Type H ECs express higher levels of PDGFA and PDGFB, TGFβ1, TGFβ3, and FGF1 relative to type L ECs, which are secreted growth factors to promote osteoprogenitor survival and proliferation. Abbreviations: ECs, endothelial cells; PDGF-BB, platelet-derived growth factor-type BB; SLIT3, slit homolog 3 protein; HIF-1α, hypoxia-inducible factor 1-alpha; VEGF, vascular endothelial growth factor; Dll 4, delta-like protein 4; RANKL, receptor activator of nuclear factor kappa-B ligand; VAO, vessel-associated osteoclast; TGFβ, transforming growth factor beta; FGF, fibroblast growth factor.

Type H vessel angiogenesis is tightly coupled with osteogenesis, which suggests the existence of molecular communications between endothelial and osteoblast cells. To date, several factors have been identified that regulate both type H vessel formation and osteogenesis. Osteoclast, osteoblast, chondrocyte, and endothelial lineage cells can secrete factors to induce proliferation of ECs, vessel assembly, and stabilization, such as PDGF-BB and SLIT3 13, 14 (**Figure 2A**). ECs can secrete factors that promote vessel assembly and stabilization and also promote bone formation, such as HIF-1α, Notch, and VEGF (**Figure 2A-B**). There are likely additional factors that remain to be elucidated.

### 3.1 Preosteoclast-derived PDGF-BB

PDGF-BB is a chemotactic and mitogenic factor in the PDGF family that is critical in promoting the migration, proliferation, and differentiation of various mesenchymal cell types, such as endothelial progenitor cells and mesenchymal stem cells to promote angiogenesis and osteogenesis 19-22. Xie et al. demonstrated that PDGF-BB enhances the formation of type H vessels and bone in the process of both bone modeling and remodeling 13. They identified preosteoclasts, the immature precursors of resorptive osteoclasts, as the main source of PDGF-BB in bone marrow and peripheral blood. This finding supports a previous report that PDGF-BB is secreted by immature, nonresorptive osteoclasts 23. Preosteoclast-derived PDGF-BB promoted the migration and differentiation of mesenchymal stem cells and bone marrow-derived endothelial progenitor cells via binding to its receptor PDGF receptor β (PDGFRβ) and triggered the mitogen-activated kinase and phosphoinositide-3 kinase-Akt signaling cascade 13. Gao et al. found that macrophage/monocytes can differentiate into periosteal tartrate-resistant acid phosphatase (TRAP^+^) mononuclear cells during bone modeling and secrete PDGF-BB, which induced periosteum-derived cell (PDC) periostin expression and recruitment to the periosteal bone surface to support osteogenesis and type H vessel formation 24. Interestingly, while this signaling mechanism was present in both young, growing mice and adult mice, two different types of PDCs were recruited. Nestin^+^ PDCs were found primarily during bone development, whereas Leptin Receptor^+^ PDCs were essential for bone homeostasis in adult mice. Altogether, preosteoclast-secreted PDGF-BB acts as an important regulator in coupling angiogenesis and osteogenesis. This finding provides evidence of communication between preosteoclasts and type H vessels and identifies a bone specific cell target to modify type H vessels **(Figure 1B and 2**).

### 3.2 SLIT3

SLIT3 is one of three SLIT ligands that were initially recognized in the central nervous system 25 where they mediate axonal guidance via receptors of the roundabout family 26. Several studies have shown that SLIT3 is expressed in other tissues beyond the nervous system and involved in additional physiological functions, such as angiogenesis 27-29 and stem cell mediation 30. In the bone, recent studies have shown that SLIT3 promotes the formation of type H vessels and bone formation 14, 31 **(Figure 1B and 2A)**.

Xu et al. found that osteoblast-derived SLIT3 promotes bone formation via its indirect effects as a proangiogenic factor to increase type H ECs numbers. Genetic deletion of *Slit3* in multiple osteoblast lineage cells (Osterix, Osteocalcin, and Dentin matrix acidic phosphoprotein 1 expressing cells) showed a reduction in type H skeletal ECs, reduced osteoblast activity and attenuated bone formation, demonstrating crosstalk between osteoblasts and type H ECs 14. Osteoblast-derived SLIT3 was further confirmed to be an essential factor in the regulation of type H vessels and bone formation using a fracture model. Defective fracture repair was noted in SLIT3-deficient mice, whereas accelerated bone fracture healing was observed in mice with a deficiency in adaptor protein Schnurri3 (a suppressor of osteoblast activity) 14. Administration of exogenous SLIT3 promoted fracture healing and prevented bone loss in the murine postmenopausal osteoporosis model by augmentation of type H vessel formation. These findings provide the first evidence of an osteoblast-derived signal that instructs type H vessels to initiate bone growth.

At nearly the same time, Kim et al. identified that osteoclast-derived SLIT3 couples bone resorption to bone formation. They reported that SLIT3 derived from mature osteoclasts stimulated the recruitment and proliferation of osteoblasts into bone remodeling sites and enhanced osteogenesis 31. Deletion of *Slit3* in Cathepsin K (Ctsk)-expressing cells in mice was associated with significantly reduced type H vessels and low bone mass, which also led to enhancement of osteoclastogenesis. These findings implicate that SLIT3 in osteoclasts may promote osteogenesis indirectly through upregulation of angiogenesis.

Overall, the primary source of SLIT3 remains debated. There are possibly multiple sources of SLIT3 in the bone marrow microenvironment. Alternatively, the genetic Cre-models utilized in both studies have overlap in cell expression, such as osteocytes 14, 31, which may be a source of SLIT3. Regardless of the cellular source, SLIT3 acts as a potent regulatory factor to induce the formation of type H vessels and bone. Administration of recombinant SLIT3 enhanced bone fracture healing and counteracted bone loss as effectively as parathyroid hormone in a mouse model of postmenopausal osteoporosis 32. Thus, drugs that target the SLIT3 signaling pathway represent a potential approach for vascular-targeted osteoanabolic therapy for treatment of osteoporosis and fracture healing.

### 3.3 HIF-1α

HIF is a transcription factor that mediates the cellular activity in response to oxygen alteration and controls physiologic and pathologic neo-angiogenesis 33, 34. HIF heterodimers consist of one of three α-subunits (HIF-1α, HIF-2α, and HIF-3α) and one β-subunit. HIF-1α expression and activity is regulated by hypoxia. High metabolic demands of osteoblasts require oxygen. Therefore, osteoblasts and nearby ECs may increase HIF-1α expression during times of relative hypoxia during osteogenesis. HIF-1α activity up-regulates VEGF expression in hypoxic tissues, making HIF-1α essential for wound regeneration and tumor vascularization 35. Similarly, HIF-1α plays a crucial role in bone formation and regeneration. Kusumbe et al. demonstrated that endothelial HIF-1α is a significant promoter of type H vessel formation in the metaphysis **(Figure 2B)**. Type H ECs expressed HIF-1α at high levels in young mice, which decreased with age and was associated with a reduction of type H ECs and age-dependent bone loss. Activation of hypoxia signaling in ECs led to an increased number of type H vessels and enhanced endochondral angiogenesis and osteogenesis 16. EC-specific deletion of HIF-1α resulted in a significant reduction of osteoprogenitors, associated with a decrease in trabecular bone formation. EC-specific inactivation of the gene for von Hippel-Lindau (*Vhl*) E3 ubiquitin ligase 36, which stabilizes HIF-1α in the endothelium, enhanced type H vessel angiogenesis and increased Osterix-positive osteoprogenitors. Osteoblasts also depend on HIF-1α signaling. Deletion of *Vhl* in osteoblasts, which results in overexpression of osteoblast HIF-1α also increased angiogenesis and osteogenesis 37. The role of HIF-1α in coupling of angiogenesis and osteogenesis, however, may be restricted to young mice, as the bone phenotype was only reported in 3-4 week old mice and disappeared at later ages 38. Administration of deferoxamine mesylate, which can promote HIF-1α activity and stability 39, significantly increased type H vessels and bone-forming osteoprogenitors and osteoblasts and thereby led to an increase in bone formation. Taken together, these findings strongly indicate that HIF-1α signaling plays a vital role in the regulation of type H vessel abundance and couples angiogenesis with osteogenesis 11, 33, 40.

### 3.4 Notch signaling

Notch and its ligand delta-like 4 transduce the signaling via cell-cell contact between ECs. Notch signaling can be regulated by blood flow. The small diameter of type H vessels confers a high flow rate which can stimulate Notch signaling, whereas decreasing blood flow decreased Notch signaling 41. In most tissues, such as murine retina, embryos, and tumors, Notch signaling restricts sprouting and mitosis in the growing vasculature and negatively modulates angiogenesis 42, 43. However, in postnatal bone, the overactivation of Notch signaling in ECs was associated with increased angiogenesis and osteogenesis 15. The opposite holds true as well. Decreased blood flow decreased Notch signaling and was associated with reduced bone volume and defective angiogenesis in aged mice 41. Increased Notch signaling through genetic manipulation of a Notch receptor inactivator (*Fbwx*) resulted in an increase in type H vessels, Runx2^+^ osteoprogenitors, and EC Noggin secretion 15. In contrast, EC-specific inactivation of Notch mutants by diminishing the essential mediator of Notch signaling (*RBPJκ*) or Delta-like 4 in postnatal bone led to a decrease in type H vessels, Noggin expression, and EC proliferation **(Figure 2B)**. Inactivation of Notch signaling was associated with decreased osteoblastic differentiation, bone formation, bone mass, and endochondral ossification. The causal relationship of Notch signaling on osteogenesis was demonstrated using recombinant Noggin protein or Notch intracellular domain in ECs, whereby the bone phenotype in Notch-signaling deficient mutants was rescued as demonstrated by similar numbers of osteoprogenitors and bone formation relative to wild type controls 15. This evidence reveals Notch signaling as a key component in the molecular crosstalk that connects angiogenesis, angiocrine signals, and osteogenesis **(Figure 2B)**.

### 3.5 VEGFA

VEGFA is considered a master regulator of angiogenesis and has been studied extensively in bone 9. VEGFA promotes EC migration and proliferation. Chondrocytes, osteoclasts, and osteoblasts can secrete VEGFA 12, 44. Transcription and secretion of VEGFA can be induced by a multitude of cytokines and transcription factors, such as HIF-1α 45, Noggin 15, PDGF-BB 13 and Runx2 46. VEGFA has multiple isoforms (VEGF_120_, VEGF_164_ and VEGF_188_ in mice and VEGF_121_, VEGF_186_, and VEGF_206_ in humans). All isoforms can bind multiple tyrosine kinase receptors, including Flt-1 (VEGFR1), Flk-1 (VEGFR2), and neuropilin 1. In endochondral bone formation, VEGF secretion from hypertrophic chondrocytes promotes vascular invasion of cartilage 12. Combined loss of VEGF_164_ and VEGF_188_ in mice results in reduction and disorganization of bone marrow vasculature, including delayed capillary invasion into hypertrophic chondroctyes, which is associated with decreased bone formation and shortened limbs 47. *In vitro* studies suggest that the temporal-spatial signaling of VEGFA and PDGF-BB is integral to vessel maturation. Persistent VEGFA signaling can impair PDGF-BB signaling through binding to Pdgrfb and inhibit perictye coverage of newly formed blood vessels 48, 49, leading to leaky blood vessels 50. Whether this temporal-spatial signaling plays an important role in type H vessel formation in bone remains unknown. High concentrations of VEGF can also increase osteoclast recruitment and bone resorption 51-54, leading to bone loss.

### 3.6 Other potential factors

Our knowledge of vascular biology in bone has been expanding, yet there are multiple candidate factors whose function in bone has not clearly been elucidated. Type H ECs express higher levels of PDGFA, transforming growth factor beta (TGFβ)1, TGFβ3, and fibroblast growth factor (FGF) 1, which are secreted growth factors that have been demonstrated to promote osteoprogenitor survival and proliferation, relative to type L ECs 16. Pericytes are also known to regulate ECs, which stabilize endothelial tube formation and facilitate maturation of the vessel through tissue- and disease-specific paracrine molecular mechanisms. PDGF-BB, Angiopoietin-1 (Ang-1) and Ang-2, TGFβ, and Sphingosine-1-phosphate (S1P) have all been implicated in mechanisms to stabilize vasculature 55. However, bone specific signaling from pericytes have not been evaluated. Recently, type H ECs have been implicated in degradation of cartilage matrix by chondroclasts during endochondral ossification through the expression of *Tnfsf11a* (the gene for receptor activator of nuclear factor kappa-B ligand (RANKL)) and *Mmp9* compared to type L ECs. Type H vessels support vessel associated osteoclasts (VOAs) through RANKL-RANK signaling which regulate anastomoses of type H vessels in return (**Figure 2A**). MMPs can regulate the extracellular matrix and modulate vessel growth or regression. MMP-9, released from type H ECs is critical for resorbing cartilage during longitudinal bone growth. MMP-2 has been implicated in changes in bone vasculature 18, 56. Further studies disrupting signaling of these growth factors/cytokines are needed to elucidate their role in coupling type H vessel angiogenesis with bone modeling and remodeling.

## 4. Type H vessels in bone disorders

In recent years, changes in type H vessels have been identified in multiple bone diseases and implicated in the pathophysiology. Drugs that target the formation and regulation of type H vessels have been demonstrated in animal disease models as potential therapeutic target of osteoarthritis, osteoporosis, delayed fracture healing, osteonecrosis, and bone metastases.

### 4.1 Osteoarthritis

**Figure 3:**
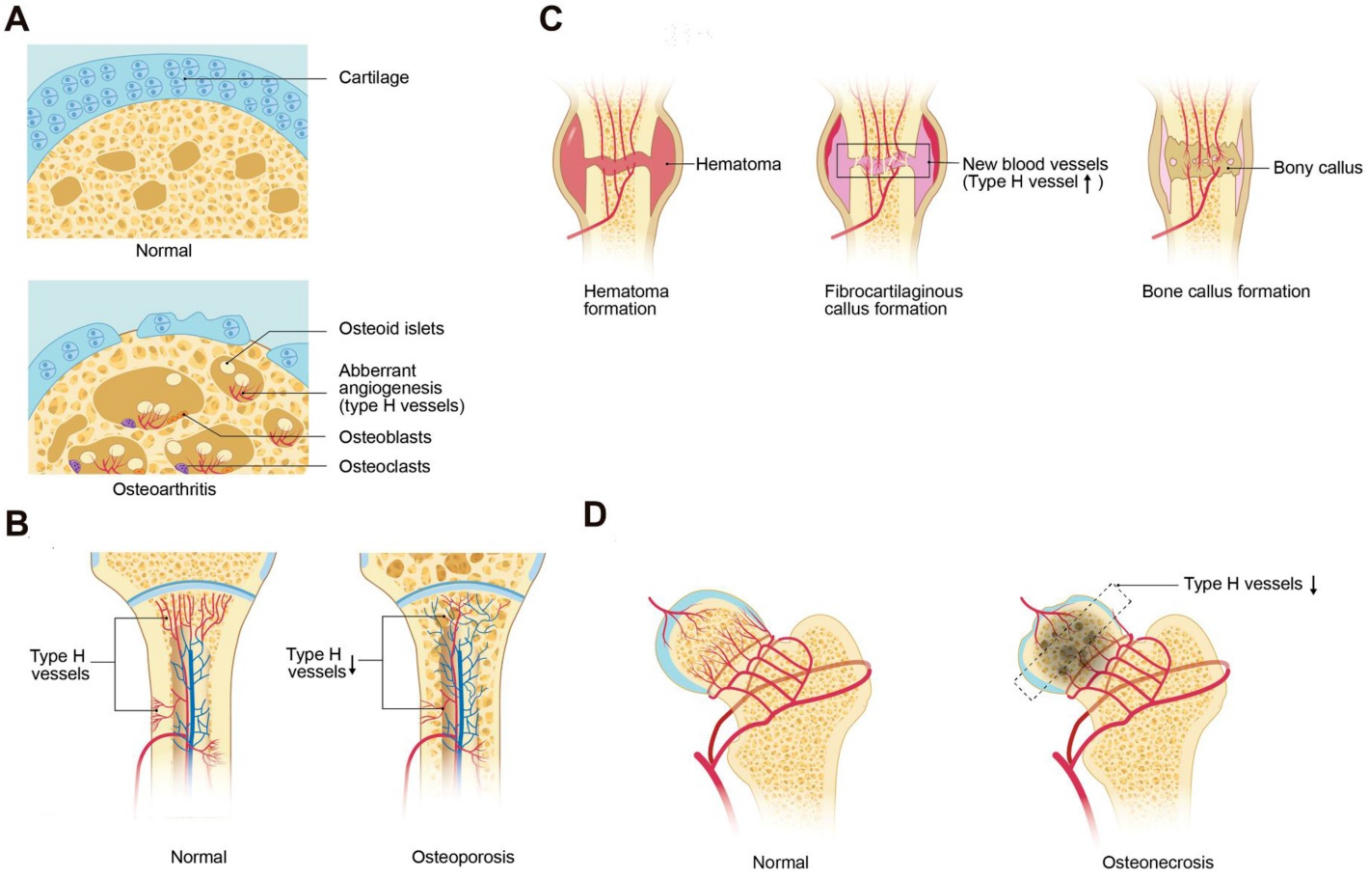
** Type H vessels involved in bone disorders. (A)** During osteoarthritis progression, type H blood vessel formation significantly increases in the subchondral bone and is associated with osteoid islet formation. The sclerotic, aberrant bone formation phenotype in the subchondral bone alters mechanical loading on the joint, contributing to cartilage degeneration. **(B)** Osteoporosis associated with either aging or GC treatment is associated with decreased type H vessels due to a decrease in angiocrine factors, such as PDGF-BB and SLIT3, which contributes to attenuated osteogenesis and net bone loss. **(C)** In fracture healing, type H vessels appear during the stage of fibrocartilaginous callus formation. Type H vessels form in the fracture callus and induce bone formation. Factors, such as SLIT3, can promote fracture healing by augmentation of type H vessel induced osteogenesis. **(D)** Femoral head osteonecrosis may result from reduced intraosseous circulation. Type H blood vessel are decreased within the subchondral bone, which may contribute to the cell death and necrosis. Abbreviations: GCs, glucocorticoids; PDGF-BB, platelet-derived growth factor-type BB; SLIT3, slit homolog 3 protein.

Osteoarthritis (OA) is a chronic disease characterized by progressive articular cartilage degeneration, articular surface and subchondral bone vascular invasion, and aberrant subchondral bone remodeling 57, 58. Lu et al. demonstrated that activation of mechanistic target of rapamycin complex (mTORC) in articular chondrocytes promoted VEGF secretion into subchondral bone and stimulated type H vessel formation. Coupling of subchondral angiogenesis with osteogenesis led to subchondral bone sclerosis and the formation of cysts and osteophytes, associated with OA progression. Suppression of chondrocyte mTORC attenuated subchondral type H vessel formation and OA progression 59. Pharmacological inhibition of mTORC by rapamycin decreased the severity of OA in mouse models, which may work in part by inhibiting pathological angiogenesis. Most recently, type H vessels were reported to digest the cartilage matrix 18, which may be another mechanism of cartilage degeneration in OA. These findings led to the question if inhibition of type H ECs may slow OA progression. Halofuginone, which can inhibit angiogenesis through indirect suppression of MMP-2 and direct inhibition of TGFβ signaling, effectively reduced aberrant type H vessels and attenuated articular cartilage degeneration and subchondral bone sclerosis 56 **(Figure 3A)**.

### 4.2 Senile/Post-Menopausal Osteoporosis

Postmenopausal osteoporosis is characterized by skeletal fragility and microarchitectural deterioration, with low bone mineral density and high risk of fractures in elderly women 60. Ovariectomized mice, mimicking postmenopausal osteoporosis, and aged mice, mimicking senile osteoporosis, have decreased type H vessels. The type H vessel decline with age are associated with decreases in angiocrine, pro-osteogenic factors and osteoprogenitors and correlate with age-related bone loss 15, 16 **(Figure 3B)**. Wang et al. reported that the abundance of type H vessels are an important indicator of bone loss in aged human subjects and in those with osteopenia 61, 62. PDGF-BB concentrations are decreased in ovariectomized mouse models likely from loss of inhibition of preosteoclast differentiation into mature osteoclasts with fewer preosteoclasts 13. Harmine, a β-carboline alkaloid, prevented bone loss via enhancement of preosteoclast PDGF-BB-induced type H vessel formation 13, 63. Additional therapeutic agents such as the Erk inhibitor, NSC-87877, which can block the fusion of preosteoclasts into mature osteoclasts and increase the production of PDGF-BB, induced the formation of type H vessels and restored bone mass in a mouse models of post-menopausal osteoporosis 64.

In conditions of aging, a notable decrease in osteoprogenitors is seen in the long bones of mice 65, which is associated with a decrease in type H vessels and a reduction in bone mass. The local delivery of tetramethylpyrazine directly induced type H vessel formation via the AMPK-mTORC-HIF-1α signaling pathway and improved bone homeostasis in aging mice 66. miR-497∼195 induced type H vessel formation via enhancement of endothelial Notch and HIF-1α activity, which provides additional therapeutic targets for the treatment of age-related osteoporosis 67.

### 4.3 Glucocorticoid-Induced Osteoporosis

Glucocorticoid-induced osteoporosis (GIO) is the most common cause of secondary osteoporosis and suppressed osteoblastic bone formation is a major contributor to bone loss 68, 69. Glucocorticoids reduce bone vasculature and blood flow 70-76. Ping et al. reported that glucocorticoids inhibit preosteoclast secretion of PDGF-BB, which led to the suppression of type H vessels and decreased osteogenesis. The cathepsin K inhibitor, L-235, was shown to prevent bone loss by inhibiting osteoclast bone resorption while maintaining preosteoclast secretion of PDGF-BB and preserving type H vessels in a young GIO mouse model 75 **(Figure 3B)**.

### 4.4 Fracture Healing

Cartilage needs to be laid down before new bone formation during fracture healing*. Thus,* cartilage resorption is an important mechanism in the process of fracture healing 77 **(Figure 3C)**. *A recent study by Romeo et al. reported that type H vessels* played a crucial role in replacing the cartilage matrix with bone during bone development and regeneration 18. This led to the question if promotion of type H vessel angiogenesis in local fracture area can enhance fracture repair. Indeed, the administration of recombinant SLIT3 effectively accelerated bone fracture healing by increasing the abundance of type H vessels in a mouse bone fracture model 14. This is a potential promising therapeutic approach, particularly in delayed union or non-unions which occur in approximately 5% of fractures 78. *Spinal fusion* is a *surgical procedure in which* two or more vertebrae are permanently connected *to treat* spinal instability 79, 80 and requires a similar mechanism to fracture repair. Low-intensity pulsed ultrasound has been reported to enhance spinal fusion by increasing type H vessel angiogenesis and the number of osteoblasts during spinal fusion 81.

### 4.5 Glucocorticoid induced osteonecrosis

GC induced osteonecrosis is a serious side effect of chronic GC use, which leads to reduced intraosseous circulation, decreased blood flow and vascular endothelial dysfunction 82, 83 **(Figure 3D)**. Weinstein et al. suggested that expression of HIF-1α and VEGF decreases in the early stage of osteonecrosis that may contribute to reduced bone vascularity 84, 85. In a glucocorticoid-treated mouse model that yields an osteonecrosis prevalence of 36%, CD31 and endomucin positive cells declined in glucocorticoid treated mice relative to vehicle. However, while treatment with LLP2A-Alendronate (a bone targeted agent that guides endogenous or exogenous mesenchymal stromal cells to bone) or parathyroid hormone (PTH) increased CD31 and endomucin cells similar to vehicle group, no change in prevalence of osteonecrosis was noted 86. Using different rodent models of osteonecrosis and PTH, other studies have reported an improvement in prevalence of osteonecrosis with a combination treatment of core decompression and PTH, including improvement in neovascularization 87. The discrepant results highlight a major limiting factor in further understanding the pathophysiology of osteonecrosis, which pertains to the preclinical animal models. Thus, it remains uncertain if blood vessel targeted therapy could alter the clinical course of osteonecrosis.

### 4.6 Bone metastasis

Bone is one of the most prevalent sites of metastasis for several cancer types 88-90. The process of the tumor cells metastasizing to bone involves multiple steps, including extravasation of the circulating tumor cells into bone. Sinusoids and sluggish blood flow within bone facilitate greater interactions between tumor cells and bone ECs 91, such that the working hypothesis of site of tumor cell metastasis to bone is more likely in the diaphysis or type L vessels rather than type H vessels. However, type H vessels supply oxygen, nutrient, cytokine, and growth factor rich blood that may promote tumor cell survival and proliferation. Manipulation of blood flow that reduces type H vessels has been associated to improve responsiveness to either radiation or chemotherapy in mouse models of metastatic breast cancer 90. Further research is on-going to define the role to type H vessels in bone metastasis.

## 5. Future perspectives

Endochondral ossification, bone modeling and bone remodeling are complicated processes that require the integrated activity of various cell types and the orchestration of multiple signaling pathways 92-95. Among these distinct cell types, a special subpopulation of ECs (type H ECs) have been shown to play a critical role in modulating the activities of osteoblasts, thereby coupling osteogenesis with angiogenesis during skeletal development and during bone regeneration and repair. This is a surprising discovery in the well-established conventional role of endothelium as a conduit system.

The description of type H vessels gives us a fuller understanding of the molecular and cellular mechanisms that underlie communications between endothelial and osteoblast cells and the interrelationship between angiogenesis and osteogenesis 16. Bone diseases and bone loss related to aging, fracture, and medication side effects are at least partly related to alterations in type H vessels. Therapeutic strategies that target type H vessels in several bone disease animal models have proven effective and successful. For example, the injection of recombinant PDGF-BB or SLIT3, which were demonstrated to be upstream factors in the regulation of type H vessel formation, were shown to prevent bone loss and accelerate bone fracture healing in mice. Likewise, the promotion of type H vessels in disease models of GIO, senile osteoporosis, spinal fusion, and prevention of type H vessel formation in an OA model has proven helpful in treating these diseases. There are likely additional key angiogenic regulatory factors that have not yet been evaluated that may play a role in bone homeostasis and disease. For example, there are no reports to date evaluating angiogenesis inhibiting factors, such as thrombospondin-1, and their effect on type H vessels in relation to bone disease.

These findings offer the interesting possibility that therapeutic strategies that target the regulatory factors of type H vessels during bone modeling and remodeling could be used to prevent disease-induced or age-related bone loss. The mechanisms that underlie several bone diseases, such as osteonecrosis, osteoarthritis and fracture nonunion, remain incompletely known and may be associated with alterations in type H vessel formation. To date, these discoveries have largely been limited to mouse disease models. Validation of the role of type H vessels in human bone disease requires further investigation.

## 6. Conclusions

Type H vessels are a newly described subtype of blood vessel in bone that play critical roles in the modulation of bone formation and repair, during both bone modeling and remodeling. Drug targeting that increases type H vessels is associated with acceleration of fracture healing and the prevention of bone loss in osteoporosis related to age or disease. The discovery of type H vessels provides new knowledge that may allow us to understand the molecular and cellular mechanisms that underlie the crosstalk between ECs and osteoprogenitors. Further research in human and animals models will offer a rational basis for the design of novel therapeutic strategies for bone repair and bone loss. Current therapies for bone disorders focus on targeting inhibition of osteoclast bone resorption or anabolic therapies stimulating osteoblast bone formation, with little regard on effect of bone vasculature. In the future, new technologies or applications that target type H vessels may represent a promising therapeutic strategy for the treatment of various bone diseases.

## Abbreviations

Emcn: endomucin
PDGF-BB: platelet-derived growth factor type BB
HIF-1α: hypoxia-inducible factor 1-alpha
HIFs: hypoxia-inducible factors
Dll 4: delta-like protein 4
ECs: endothelial cells
OA: osteoarthritis
SLIT3: slit homolog 3 protein
mTORC: mechanistic target of rapamycin complex
Vhl: von hippel-lindau
VEGF: vascular endothelial growth factor
TGFβ: transforming growth factor beta
FGF: fibroblast growth factor
Ang-1: angiopoietin-1
S1P: sphingosine-1-phosphate
MMP-9: matrix metalloproteinase-9
VAOs: vessels associated osteoclasts
GCs: glucocorticoids
Ctsk: cathepsin K
PTH: parathyroid hormone
GIO: glucocorticoid-induced osteoporosis
ON: osteonecrosis
